# PDIA iminosugar influence on subcutaneous *Staphylococcus aureus* and *Pseudomonas aeruginosa* infections in mice

**DOI:** 10.3389/fcimb.2024.1395577

**Published:** 2024-07-31

**Authors:** Łucja Kozień, Aleksandra Policht, Piotr Heczko, Zbigniew Arent, Urszula Bracha, Laura Pardyak, Agnieszka Pietsch-Fulbiszewska, Estelle Gallienne, Piotr Piwowar, Krzysztof Okoń, Anna Tomusiak-Plebanek, Magdalena Strus

**Affiliations:** ^1^ Department of Bacteriology, Ecology of Microbes and Parasitology, Faculty of Medicine, Jagiellonian University Medical College, Krakow, Poland; ^2^ Center of Experimental and Innovative Medicine, University Centre of Veterinary Medicine JU-UA, University of Agriculture in Krakow, Krakow, Poland; ^3^ Institut de Chimie Organique et Analytique (ICOA), UMR 7311, Université d'Orléans & CNRS, Orléans, France; ^4^ Faculty of Electrical Engineering, Automatics, Computer Science and Biomedical Engineering, AGH University of Science and Technology, Kraków, Poland; ^5^ Department of Bacteriology, Ecology of Microbes and Parasitology, Faculty of Medicine, Jagiellonian University Medical College, Kraków, Poland

**Keywords:** antibiofilm drug, wound infection, *Staphylococcus* infection, *Pseudomonas* infection, PDIA iminosugar

## Abstract

**Introduction:**

Biofilm-associated infections persist as a therapeutic challenge in contemporary medicine. The efficacy of antibiotic therapies is ineffective in numerous instances, necessitating a heightened focus on exploring novel anti-biofilm medical strategies. Among these, iminosugars emerge as a distinctive class of compounds displaying promising biofilm inhibition properties.

**Methods:**

This study employs an *in vivo* wound infection mouse model to evaluate the effectiveness of PDIA in treating biofilm-associated skin wound infections caused by *Staphylococcus aureus* and *Pseudomonas aeruginosa*. Dermic wounds in mice were infected with biofilm-forming strains, specifically *S. aureus* 48 and *P. aeruginosa* 5, which were isolated from patients with diabetic foot, and are well-known for their strong biofilm formation. The subsequent analysis included clinical, microbiological, and histopathological parameters. Furthermore, an exploration into the susceptibility of the infectious strains to hydrogen peroxide was conducted, acknowledging its potential presence during induced inflammation in mouse dermal wounds within an *in vivo* model.

**Results:**

The findings revealed the efficacy of PDIA iminosugar against the *S. aureus* strain, evidenced by a reduction in bacterial numbers within the wound and the inflammatory focus.

**Discussion:**

This study suggests that PDIA iminosugar emerges as an active and potentially effective antibiofilm agent, positioning it as a viable treatment option for staphylococcal infections.

## 1 Introduction

Due to its multifaceted resistance to multiple drugs, biofilm formation presents a considerable and persistent threat in human bacterial and mycotic infections, contributing to chronic infections that are challenging to treat. Therefore, research on novel strategies to prevent and/or treat pathogen biofilm formation is one of the most expanding fields in experimental medicine. Among the most challenging objectives, developing a viable technique that selectively targets adhesive properties without compromising bacterial viability is imperative (Srinivasan et al., 2021). Furthermore, bacteria and yeasts produce biofilms on different indwelling medical devices such as implants, vascular grafts, heart valves, intrauterine devices, pacemakers, prosthetic joints, catheters, sutures, and contact lenses (Kannappan et al., 2017).

*Staphylococcus aureus* and *Pseudomonas aeruginosa* are among the most prevalently defined etiological agents causing a range of severe nosocomial, chronic, antibiotic-resistant, and biofilm-associated infections (Mulcahy et al., 2014; Cheung et al., 2021). They exhibit diverse virulence factors encompassing the synthesis of pathogenic toxins (both proteinaceous and non-proteinaceous molecules) along with genetic determinants facilitating their adept colonization and sustained persistence within a host organism (Tolker-Nielsen, 2014; Graf et al., 2019). Among countless defensive strategies various bacteria employ to counter unfavorable external conditions, forming a biofilm stands out as a particularly efficacious protective mechanism (Dos Santos et al., 2018). The strain-dependent nature of the bacteria in question significantly influences the multiplicity of virulent factors and pathogenicity. For instance, amidst tissue inflammation, phagocytic cells of the immune system, including neutrophils, undergo a respiratory burst upon encountering antigens from infectious microorganisms. This event culminates in the release of reactive bactericidal products stemming from aerobic metabolism, such as singlet oxygen (1O2) or hydrogen peroxide (H_2_O_2_) (Clark, 1999). Certain bacteria, such as *P. aeruginosa* and *S. aureus*, can mitigate the harmful impacts of H_2_O_2_ generated by host cells. In addition to biofilm formation, bacteria employ a mechanism to shield themselves from oxidative stress by producing a diverse array of enzymes, including catalase, superoxide dismutase (SOD), and reductases. However, this capability is strain-dependent (Allaoui et al., 2009). Consequently, strains capable of biofilm production yet failing to initiate its formation prior to the onset of host inflammation may encounter challenges in survival and may be hindered in their ability to instigate a severe infection.

Amidst the manifold therapeutic strategies recently documented for biofilm inhibition encompassing diverse mechanisms and categorized into various classes as outlined by (Srinivasan et al., 2021), only the dissolution of exopolysaccharide (EPS) structures within the biofilm is explicitly highlighted. Notably, a specific class of iminosugars can inhibit biofilm synthesis, potentially through interaction with the glucosyltransferase enzyme involved in EPS synthesis (Ren et al., 2016). The prototype of this group was already described in 2008 by Islam as an anti-adherence activity of mulberry leaves. The active substance appeared to be 1-deoxynojirimycin, which inhibited EPS production by *Streptococcus mutans* (Islam et al., 2008). Our group described several synthetic iminosugar derivatives actively inhibiting early biofilm formation by *P. aeruginosa* but not influencing bacterial reproduction (Strus et al., 2016). More recently, except for its anti-biofilm activity, N-nonyloxypentyl-L-DNJ iminosugar enantiomer was reported to be a molecule displaying high antibacterial properties against *S. aureus* (De Gregorio et al., 2020). Last year, we published a paper on beta-1-C-propyl-1,4-dideoxy-1,4-imino-L-arabinitol (PDIA iminosugar) which inhibited early biofilm production of *Enterobacter* spp., *P. aeruginosa*, *Enterococcus* spp. and *S. aureus* at 8 and 24 h, and *Klebsiella* spp., *Acinetobacter* spp. and *S. epidermidis* in 24 h. In addition, we found that the same iminosugar inhibited mature biofilm formation of *P. aeruginosa* in 32 h and of *Acinetobacter* spp., *S. aureus*, and *S. epidermidis* at 48 h. PDIA at a concentration of 0.9 mM caused no growth inhibition of the tested bacteria (Kozień et al., 2022).

The current investigation sought to assess the *in vivo* effectiveness of PDIA in an animal model simulating experimental subcutaneous wound infection induced by *S. aureus* and *P. aeruginosa*. Additionally, endeavors were made to observe distinct trajectories of local inflammatory processes provoked by *S. aureus* and *P. aeruginosa* within tissues during experimental skin infections. Moreover, the infectious strains’ susceptibility to H_2_O_2_ was assessed to outline potential environmental factors encountered in the *in vivo* investigation.

## 2 Materials and methods

### 2.1 Bacterial strains and growth conditions

Two bacterial strains (*S. aureus* 48 and *P. aeruginosa* 5) were deliberately chosen based on their demonstrated high biofilm-forming proficiency and the biofilm susceptibility to the inhibitory properties of PDIA iminosugar (Kozień et al., 2022). Both strains are integral components of the bacterial strains’ repository at the Department of Microbiology, Jagiellonian University Medical College, originating from patients afflicted with diabetic foot infections (*P. aeruginosa* 5) and chronic otitis media (*S. aureus* 48). The taxonomic identities of these strains were verified through mass spectrometry (MALDI-TOF MS Biotyper, Bruker Scientific LLC, Billerica, MA, USA) following the manufacturer’s guidelines.

To initiate bacterial cultures, an inoculum was generated by subjecting glass beads coated with bacteria from cryogenically preserved pure cultures to incubation in 10 mL of Tryptic Soy Broth (TSB) (Beckton Dickinson, Franklin Lakes, NJ, USA) at 37°C for 24 hours. Subsequently, to ensure strain purity, 10 µL aliquots of the cultures were streaked onto Columbia Agar (Biomaxima, Poland) and incubated similarly. This process was iterated three times to attain bacterial populations of heightened viability.

For further experimentation, a single colony from 24-hour pure culture plates was introduced into 10 mL of TSB broth using a sterile loop, thoroughly mixed, and incubated at 37°C for 24 h. Post-incubation, a serial dilution methodology in TSB broth generated a bacterial suspension of a 1.0 x 10^5^ colony-forming units of bacteria per milliliter (CFU/mL) density (Sutton, 2011). These meticulously prepared suspensions of 1.0 x 10^5^ CFU/mL density were utilized in subsequent stages of the experimental protocol.

### 2.2 Iminosugar

The iminosugar derivative under investigation (PDIA, beta-1-C-propyl-1,4-dideoxy-1,4-imino-L-arabinitol) was synthesized at the Institute of Organic and Analytical Chemistry, University of Orleans and CNRS, France. Our preceding study presented its chemical structure (Kozień et al., 2022). In the experiments, a solution of iminosugar at a concentration of 1 mM was employed. This concentration was chosen based on previous *in vitro* experiments, described by Kozień et al. (2022), in which 0.9 mM concentration appeared to be effective. Thus, the dose of 1 mM was used locally *in vivo*. To obtain this concentration, PDIA was dissolved in DMSO (Sigma Aldrich, USA) to a concentration of 100 mM. Next, to reduce the DMSO toxicity, it was diluted in sterile water reaching the final concentration of 1mM PDIA in 1% DMSO.

### 2.3 Animals

All experiments with animals were performed following the Guide for the Care and Use of Laboratory Animals published by the US National Institutes of Health and have received approval under resolution 6/2022 from the Second Local Institutional Animal Care and Use Committee (IACUC) in Kraków.

Six groups of male and female BALB/C mice (Animal Facility of Clinical Immunology and Transplantation Department of the Pediatrics Institute in the Jagiellonian University Medical College) aged 6-10 weeks were employed. Mice were divided into following groups: procedure control (9 mice); iminosugar control (8 mice); *S. aureus* 48 (SA 48) infection (10 mice); SA 48 infection with iminosugar (8 mice); *P. aeruginosa* 5 (PAR 5) infection (8 mice) and PAR 5 infection with iminosugar (8 mice).

### 2.4 Experimental wound infection

The model of subcutaneous infection was modified as previously described (Liu et al., 2019). Following the induction of general anesthesia via an intraperitoneal administration of a combined dose of ketamine (Vetaketam, VET-ARGO, Poland) and xylazine (Vetaxyl, VET-ARGO, Poland) at 50 mg/kg (ketamine) and 5 mg/kg (xylazine), a 10-millimeter incision was meticulously made on the dorsal region of each mouse subject. Subsequently, the skin was delicately dissected to create a subcutaneous pouch. The resultant wounds were then deliberately subjected to infection with designated bacterial strains or left uninfected, serving as the control groups.

Distinct bacterial strains were employed for the infected cohorts. In the instances of the infected groups, a 50 µl suspension of the selected bacterial strains (boasting a density of 1 x 10^5^ CFU/mL) was meticulously introduced into the formed subcutaneous pouch. To gauge the *in vivo* efficacy of the PDIA iminosugar being tested on the two infection groups (each with distinct strains), a 500 µl solution of 1 mM iminosugar was systematically injected into the subcutaneous pouch containing the bacterial suspension. The control group of animals received the same volume of sterile saline. Subcutaneous meloxicam (Melovem, Dopharma B.V., The Netherlands) was administered as an analgesic at 5 mg/kg.

The groups were arranged and divided, as shown in **Table 1**.

**Table 1:** Experimental groups.

Group name	Number of mice in the group (n)	Infectious strain	Procedure
Control	9	–	subcutaneous incision
Control + I	8	–	subcutaneous incision + PDIA iminosugar
SA48	10	*S. aureus* 48	subcutaneous incision + *S. aureus*
SA48 + I	8	*S. aureus* 48	subcutaneous incision + *S. aureus* + PDIA iminosugar
PAR5	8	*P. aeruginosa* 5	subcutaneous incision + *P. aeruginosa*
PAR5 + I	8	*P. aeruginosa* 5	subcutaneous incision + *P. aeruginosa* + PDIA iminosugar

Control: procedure control (subcutaneous incision alone), Control + I: iminosugar control (subcutaneous incision with insertion of PDIA iminosugar), SA 48: control of S. aureus infection (subcutaneous incision with insertion of S. aureus strain 48), SA48 + I: S. aureus infection with iminosugar (subcutaneous incision with insertion of S. aureus strain 48 and PDIA iminosugar), PAR5: control of P. aeruginosa infection (subcutaneous incision with insertion of P. aeruginosa strain 5) and PAR 5 + I: P. aeruginosa infection with iminosugar (subcutaneous incision with insertion of P. aeruginosa strain 5 and PDIA iminosugar).

After bacterial inoculation and iminosugar injection, each wound was carefully sutured, and the animals were maintained for three additional days under conditions consistent with those during the adaptation period. Throughout this interval, comprehensive clinical assessments were conducted, including meticulous evaluations of wound healing and the systematic monitoring of edema, redness, and exudate appearance, strictly adhering to the procedures delineated in section 2.5. Following a three-day interval, euthanasia of the mice was carried out through the dislodgement of the cervical vertebrae after anesthesia induction via isoflurane (Isotek, Laboratorios Karizoo S.A., Spain) administered at a dosage of 1000 mg/g (Marquardt et al., 2018). Subsequently, a necropsy was conducted, during which tissues and organs were harvested for microbiological and histopathological examinations.

### 2.5 Clinical evaluation scale

The assessment of wound healing throughout the 3-day experimental period involved the application of a clinical evaluation scale. The surgical wound was appraised through a modified version of the NERDS and STONEES scales, initially designed for evaluating wound infections in human subjects (Woo and Sibbald, 2009). Particular attention was directed towards four discernible parameters — healing, exudate, edema, and redness — each exhibiting varying degrees of intensity. The observations were systematically categorized utilizing the ensuing scale:

- **Healing:** 0 - indicative of normal healing, 1 - indicative of wound dehiscence
- **Exudate:** 0 - representing a dry wound, 1 - signifying a moist wound with slight exudate, 2 - indicative of a moist wound with abundant exudate
- **Edema:** 0 - suggesting an absence of edema, 1 - denoting a significant level of edema, 2 - representing a substantial presence of edema
- **Redness:** 0 - indicative of the absence of redness, 1 - denoting the presence of redness

### 2.6 Microbiological analysis

On day three, the specimens obtained from each mouse during the post-surgery necropsy comprised wound swabs, intracardial blood samples (ranging in volumes from 300 to 1000 µl), and spleens. After collection, the wound swabs, blood samples, and spleens were immersed in 5, 0.5, and 1.5 milliliters of TSB broth. The spleens underwent homogenization using a sterile mortar. Following meticulous mixing, NaCl-based decimal serial dilutions were prepared, and the samples were quantitatively inoculated onto the respective agar media: Columbia Agar (BioMaxima, Poland), MacConkey Agar (BioMaxima, Poland), and Sabouraud Dextrose Agar (BioMaxima, Poland). The inoculated agar plates were then incubated at **37°C** for **24** h (Lagier et al., 2015).

#### 2.6.1 Isolation of *S. aureus* 48 and *P. aeruginosa* 5 strains from mouse specimens based on drug resistance profiles

During microbiological testing, our paramount objective was to detect *S. aureus* 48 and *P. aeruginosa* 5 strains within the infection groups’ wounds. To attain this goal, we executed a comparative scrutiny of the drug susceptibility profiles characterizing the subjected strains. Colonies of *Staphylococcus* spp. were cultured on Columbia medium, while those of *Pseudomonas* spp. were cultivated on MacConkey medium. Subsequently, colonies were sub-cultured onto agar plate sectors and incubated at 37°C for 24 h. Following incubation, a 0.5 McFarland saline suspension was crafted from each sector and introduced into Mueller-Hinton II Broth (Biomaxima, Poland) via a sterile swab. Antibiotic discs (OXOID, UK), precisely detailed in **Tables 2**, **3**, were affixed to the surfaces of the pre-inoculated plates of Mueller-Hinton II Agar (Biomaxima, Poland) and subjected again to a 24 h incubation period at 37°C. After incubation periods, the measured growth inhibition zones (in millimeters) facilitated the derivation of drug resistance patterns. Establishing the identity of isolates from wounds necessitated a thorough juxtaposition of the acquired drug resistance profiles with the reference test strains, as delineated in **Tables 2**, **3**. This methodical comparative analysis facilitated the accurate discernment of the isolates correlated with the specified test strains and has already been used in our other study (Tomusiak-Plebanek et al., 2018).

**Table 2:** Drug resistance patterns of *S. aureus* 48 strain.

Strain tested	The size of the inhibition zone [mm] for individual antibiotics				
	FOX (30µg)	DA (2 µg)	E (15 µg)	CN (10 µg)	SXT (25 µg)
*S. aureus* 48	R	R	R	21	30

R, Resistance; FOX, Cefoxitin; DA, Clindamycin; E, Erythromycin; CN, Gentamycin; SXT, Sulfamethoxazole/Trimethoprim.

**Table 3:** Drug resistance patterns of *P. aeruginosa* 5 strain.

Strain tested	The size of the inhibition zone [mm] for individual antibiotics				
	IMP (10 µg)	ATM (30 µg)	CIP (5 µg)	TOB (10 µg)	CAZ (10 µg)
*P. aeruginosa* 5	25	23	31	21	19

IMP, Imipenem; ATM, Aztreonam; CIP, Ciprofloxacin; TOB, Tobramycin; CAZ, Ceftazidime.

### 2.7 Histological analysis

Tissue sections encompassing the epidermis, dermis, and panniculus carnosus muscle were procured from each mouse subject for subsequent histopathological scrutiny. These tissue samples were placed in tubes containing 10% formalin (Chempur, Poland). Post-fixation, the skin tissues underwent processing in a state-of-the-art tissue processor (Thermo Shandon Limited EXCELSIOR AS, UK) and were embedded in paraffin (Thermo Shandon Limited HISTOSTAR, UK). The resultant paraffin blocks were precision-cut into 3-µm sections using a microtome (LEICA Histocore AUTOCUT, Germany) and stained with hematoxylin and eosin (SAKURA PRISMA E2S, Netherlands), followed by cover-slipping with a film (SAKURA FILM COVERSLIPPER, Netherlands). Histologic evaluation was undertaken by a certified histopathologist utilizing an Olympus BX53 microscope, with observations made under magnifications of up to 400 x. The structural attributes of the specimens were methodically described, encompassing considerations of tissue composition, inflammatory intensity, the constitution of inflammatory infiltrate, the presence of granulation tissue, and any indications of ulceration. The quantification of neutrophilic infiltrate, chronic inflammatory cells, and granulation tissue was categorized on a scale of 0 to 3 (Klopfleisch, 2013) where:

- 0 - absence of the given feature
- 1 - mild intensity
- 2 - moderate intensity
- 3 - severe intensity

The cumulative parameters for each mouse subject were aggregated and subjected to rigorous statistical analysis.

### 2.8 Hydrogen peroxide’s *in vitro* effect on *S. aureus* 48 and *P. aeruginosa* 5 strains

To ascertain the potential impact of H_2_O_2_ on pathogenic strains of *S. aureus* 48 and *P. aeruginosa* 5 under *in vivo* conditions, an empirical investigation was conducted to scrutinize the time-based dynamics of the test bacteria population in the presence of H_2_O_2_. The experiment was conducted based on our previously described procedure, incorporating certain modifications (Strus et al., 2006). Sequential dilutions of 3% H_2_O_2_ (APTEO, Poland) in aseptic distilled water were prepared, yielding final concentrations spanning from 110 mM to 0.6 mM. *S. aureus* 48 and *P. aeruginosa* 5 strain cultivation adhered to the methodology expounded in Section 2.1.

A singular colony from 24 h pure culture plates was introduced into 10 mL of TSB broth via a sterile loop, comprehensively mixed, and subjected to a 24 h incubation at 37°C.

Following incubation, a serial dilution approach in TSB broth solution was employed to generate a bacterial suspension with a density of 1.0 x 10^5^ CFU/mL, mirroring conditions analogous to *in vivo* scenarios. These suspensions served as the foundation for subsequent experimental phases.

The experimentation transpired within sterile glass tubes. Extracting 700 µl from 1.0 x 10^5^ CFU/mL bacterial suspensions, each sample was enriched with 100 µl of the relevant H_2_O_2_ concentration, ensuring a final sample volume encompassing H_2_O_2_ concentrations ranging from 0.6 mM to 110 mM. Nine concentrations were systematically examined for each strain. The control cohort incorporated an equivalent volume of bacterial culture, supplemented with 100 μl of distilled water. The assays were performed in duplicate.

Both the test and control tubes underwent a 6 h incubation period at 37°C. At intervals of 0, 1, 4, and 6 h, 100 μl from each suspension was harvested, subjected to serial dilutions on tryptone soy agar (TSA, Biomaxima, Poland), and incubated again at 37°C for 24 h to ascertain bacterial quantification.

### 2.9 Statistical analysis

The statistical analysis was executed within the R environment (R Core Team, 2019), leveraging non-parametric tests due to the failure to meet assumptions requisite for parametric tests. Specifically, the Wilcoxon rank sum test was employed to evaluate disparities in medians between groups. P values ≤0.05 were considered statistically significant. The graphical representation of the findings was crafted utilizing the OriginLab software (OriginPro 2021).

## 3 Results

The comparison among distinct groups of animals was conducted through three autonomous assays: clinical observations, quantification of inoculated bacteria in dissected tissues, and microscopic scrutiny of tissue alterations.

### 3.1 Clinical observations

No fatalities among the mice were documented in any experimental group. Examination of the data gleaned from clinical observations conducted on infected, treated, and control animals revealed discrepant outcomes. The detailed results can be referenced in the **Supplementary Materials** (**Supplementary Figure 1**).

### 3.2 Microbiology

Bacteria numbers representative of the test strains in animal tissues obtained from mouse wound swabs 72 h after wound formation/infection are outlined in **Table 4**. Notably, no bacterial presence was detected in blood or spleen samples collected from the mice. The comparison of the resistance patterns of the wound isolates with the injected bacteria confirmed that they were identical as judged on their resistance patterns.

**Table 4:** The number of bacteria in animal tissues at 72 hours of the experiment in Control groups and groups infected with *Staphylococcus aureus* 48 and *Pseudomonas aeruginosa* 5.

Group name	Control	Control + I	SA 48	SA 48 + I	PAR 5	PAR 5 +I
Mouse number	The number of bacteria present in wound swabs collected from mice [CFU/mL]		The number of inoculated *Staphylococcus aureus* 48 bacteria in wound swabs collected from mice [CFU/mL]		The number of inoculated *Pseudomonas aeruginosa* 5 bacteria in wound swabs collected from mice [CFU/mL]	
1	ND	ND	4.20E+04	3.50E+04	1.50E+01	1.00E+01
2	ND	ND	3.00E+04	6.00E+04	2.50E+01	ND
3	ND	ND	2.00E+04	1.50E+04	ND	ND
4	ND	ND	6.00E+05	5.55E+04	3.00E+02	1.50E+01
5	ND	ND	4.00E+05	1.00E+04	ND	ND
6	ND	ND	6.00E+03	1.00E+03	ND	1.25E+03
7	ND	ND	1.60E+04	5.00E+00	2.00E+01	ND
8	ND	ND	1.00E+04	1.00E+02	ND	1.50E+02
9	ND		4.00E+04			
10			4.00E+04			
**average**	**ND**	**ND**	**1.20E+05**	**2.21E+04**	**4.50E+01**	**1.78E+02**
**SD**			**2.05E+05**	**2.48E+04**	**1.40E+02**	**5.99E+02**
**p < 0.05**			**0.0411**			

ND, not detectable.

The bold values indicate the means obtained from the analysis of all examined mice in respective groups, along with standard deviations.

**Figure 1:**
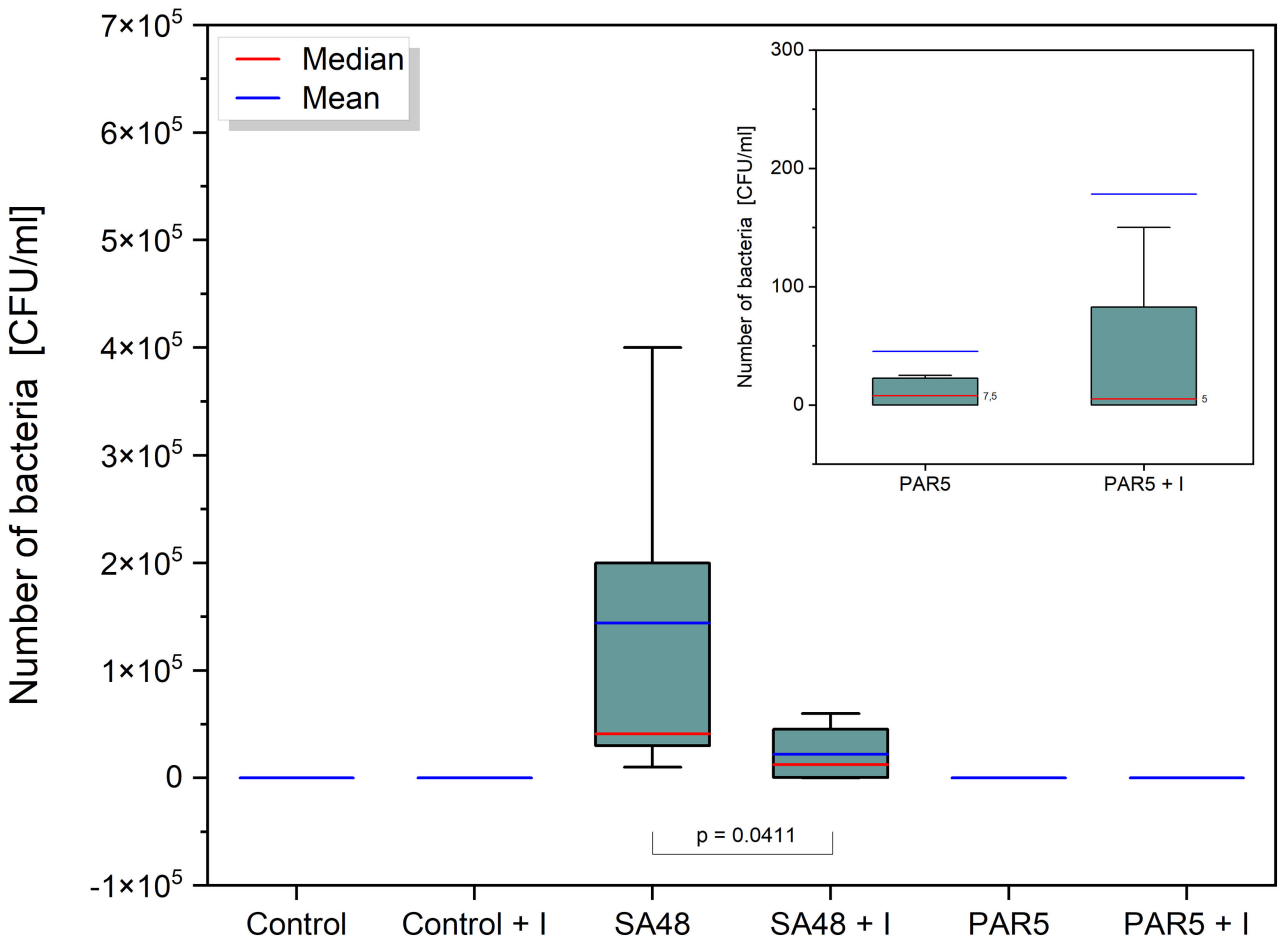
Box plot showing the distribution [CFU/mL] of *S. aureus 48* bacteria in the SA 48 and SA 48 + I groups, and *P. aeruginosa 5* bacteria in the PAR5 and PAR 5 + I groups, extracted from wound swabs taken from mice 72 h after wound formation/infection. The graph indicates statistical significance obtained after comparing all results.

The application of the PDIA iminosugar exhibited a noteworthy impact, significantly inhibiting the proliferation of the *S. aureus* 48 strain in mice tissues (p=0.0411) over three days (72 h) compared to control conditions. The comprehensive results are visually presented in **Figure 1**. Notably, such inhibitory effects were not evident in the groups of mice infected with *P. aeruginosa* 5. Furthermore, in contrast to the groups infected with *P. aeruginosa* 5, those infected with *S. aureus* 48 demonstrated a markedly elevated bacterial number.

### 3.3 Histopathology

**Figure 2:**
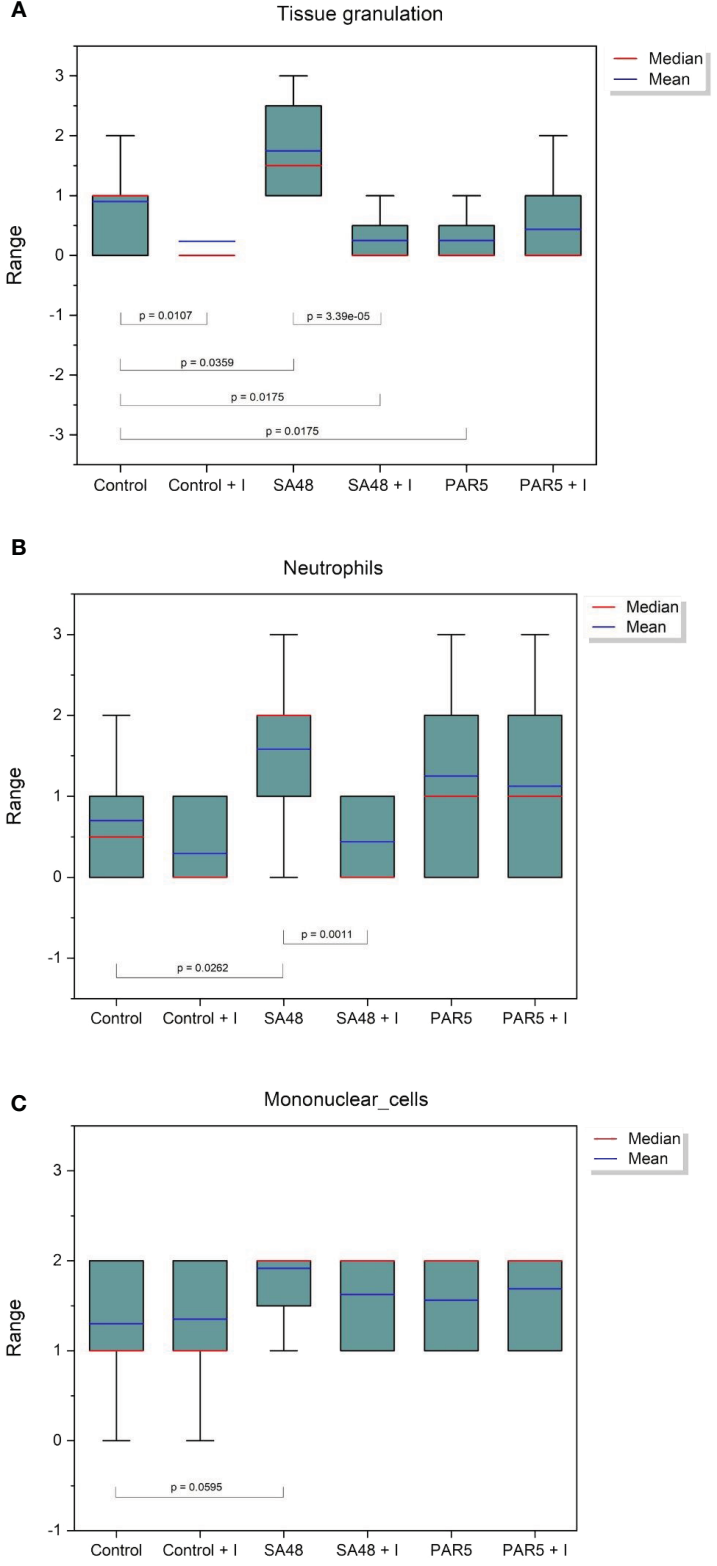
Box plot showing the distribution of histopathological findings for: **(A)** Tissue granulation, **(B)** Neutrophil inflammatory cells, and **(C)** Mononuclear cells for each study group. Statistical significance is indicated on the graph.

**Figure 3:**
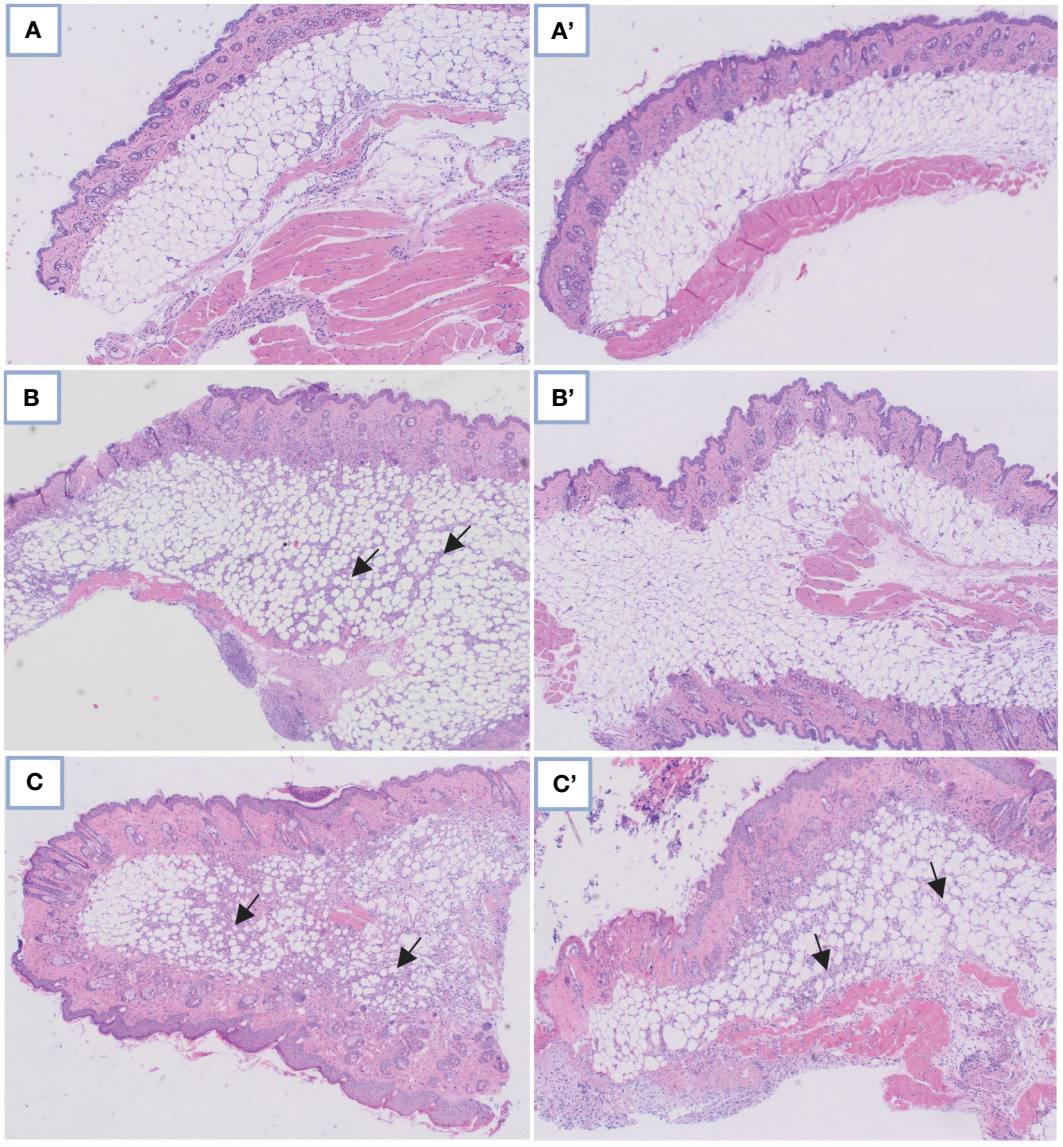
Comparison of microscopic images in control skin tissue: **(A)** Without and A’ with PDIA iminosugar administration; in the *S. aureus*-infected skin tissue: **(B)** Without and B’ with PDIA iminosugar administration; in the *P. aeruginosa*-infected skin tissue: **(C)** Without and C’ with PDIA iminosugar administration. Histopathology was performed using an upright microscope at 400× magnification. The black arrows indicate an inflammatory focus exacerbation.

The observations regarding the quantities of *S. aureus* bacteria in infected mice were corroborated through histopathological examination. Significant differences were identified concerning the reduction in tissue granulation (p=3.3908 x 10^-5^) and the decrease in neutrophil inflammatory cell accumulation within tissues (p=0.0011). These findings are visually depicted in **Figures 2**, **3**.

No comprehensible association was identified within groups afflicted with *Pseudomonas* infection. Statistically noteworthy distinctions were manifested in tissue granulation (p=0.0359) and the aggregation of inflammatory neutrophil cells (p=0.0262) within the *S. aureus* 48 group when juxtaposed against the control group. These disparities substantiate staphylococcal infections’ considerably more severe trajectory than *Pseudomonas* infections, where such distinctions remained elusive.

The utilized iminosugar concentration exhibited a potential mitigating effect on the inflammatory response within subcutaneous pocket tissues, as evidenced by the diminished tissue granulation in the iminosugar-treated control group relative to the iminosugar-free control group (p=0.0107).

Clinical observations of both control and test subjects failed to yield conclusive data, perhaps attributed to the employed technique, wherein a significant portion of the inflammatory process was concealed within the pockets beneath the animals’ skin.

### 3.4 Hydrogen peroxide impact on *S. aureus* 48 and *P. aeruginosa* 5

The precise viable bacterial counts of *S. aureus* 48 and *P. aeruginosa* 5 following exposure to varying concentrations of H_2_O_2_ (110 mM, 55 mM, 27.65 mM, 14 mM, 6.8 mM, 3.5 mM, 1.8 mM, 0.88 mM, and 0.59 mM) at designated time points (0 h, 1 h, 4 h and 6 h) are detailed in the **Supplementary Materials**.

**Figure 4:**
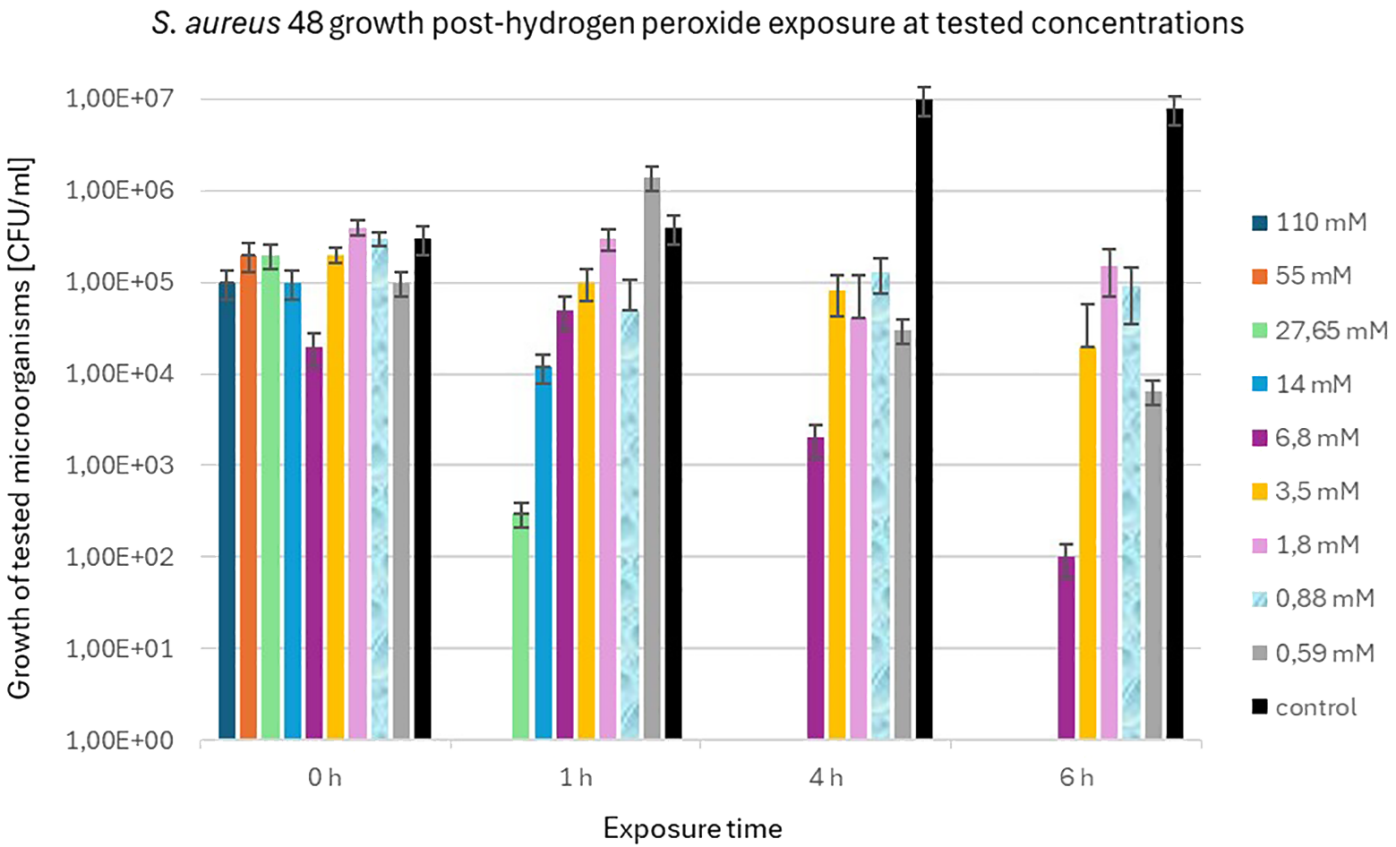
Population counts [CFU/mL] of *S. aureus* 48 during 6-hour exposures to different H_2_O_2_ concentrations versus unexposed controls.

*S. aureus* 48 demonstrated high resistance to high concentrations of H_2_O_2_, as illustrated in **Figure 4**. After 6 h of exposure, an decrease in the bacterial population of *S. aureus* 48 was noted at the following concentrations of H_2_O_2_: 6.8 mM, 3.5 mM, 1.8 mM, 0.88 mM, and 0.59 mM.

**Figure 5:**
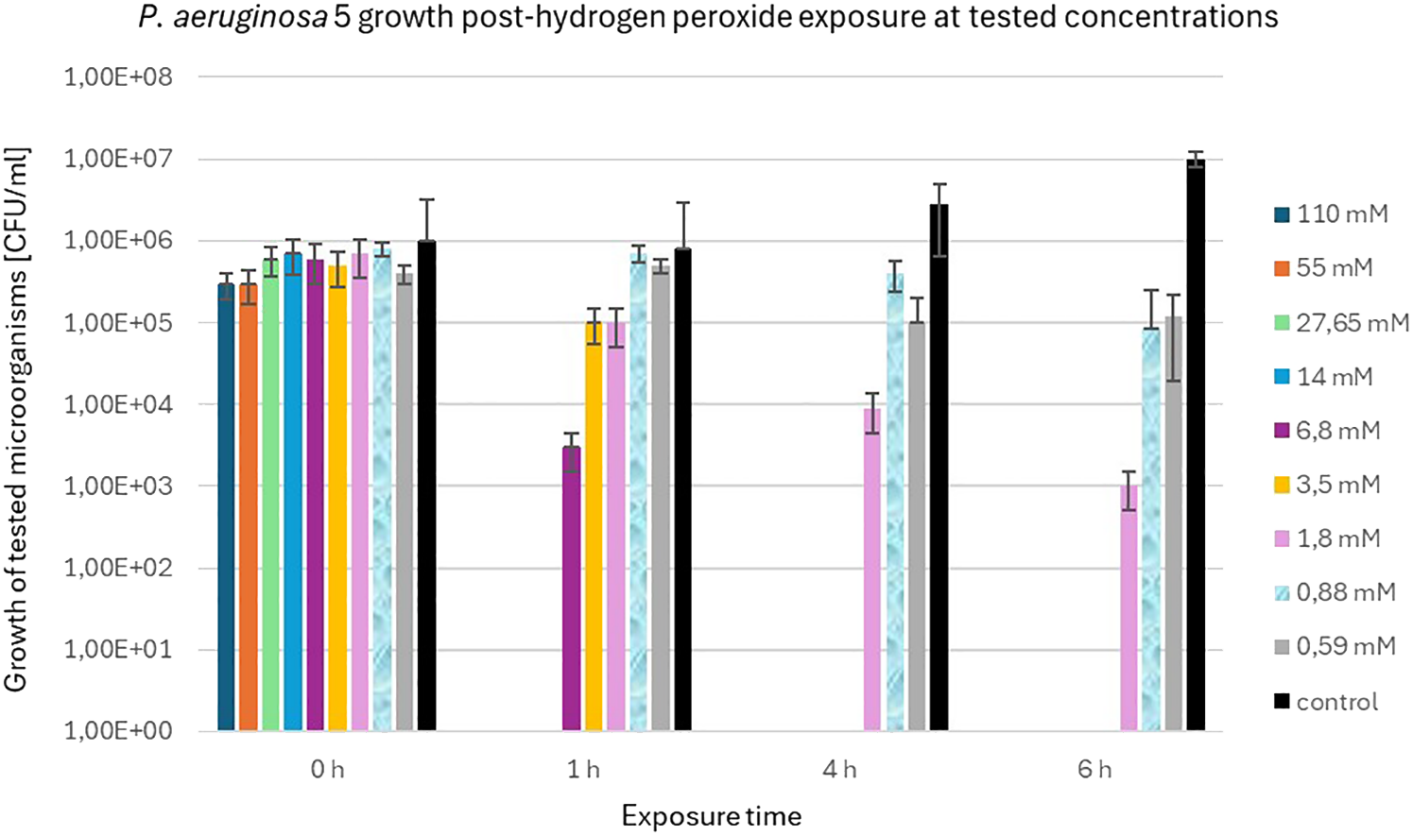
Population counts [CFU/mL] of *P. aeruginosa* 5 during 6-hour exposures to different H_2_O_2_ concentrations versus unexposed controls.

In contrast to *S. aureus* 48, *P. aeruginosa* 5 exhibited markedly diminished resistance to elevated concentrations of H_2_O_2_. **Figure 5** illustrates the population dynamics of *P. aeruginosa* 5 following a 6 h exposure to H_2_O_2_ concentrations of 1.8 mM, 0.88 mM, and 0.59 mM. Notably, it is observed that *S. aureus* 48 demonstrated growth under identical experimental conditions, extending to H_2_O_2_ concentrations of 6.8 mM and 3.5 mM.

## 4 Discussion

While numerous publications have explored biofilm inhibitors, only a limited number have investigated the efficacy of anti-biofilm structures in *in vitro* studies. Recently, a surge in studies has been reported, employing diverse experimental designs and host organisms ranging from the nematode *Caenorhabditis elegans* to rodents (O’Loughlin et al., 2013; Luo et al., 2017; Jordan et al., 2022).

Experiments conducted on nematodes offer insight into fundamental relationships, specifically their survival in the presence of bacterial pathogens with or without an antibiofilm compound treatment. Despite claims of relevance to bacterial proliferation in the nematode gut (Utari and Quax, 2013; Luo et al., 2017), this model is somewhat distant from the complex *in vivo* conditions encountered in infected wounds.

Contrastingly, investigations involving mouse subjects afford a more comprehensive exploration, encompassing not only the assessment of post-bacterial load morbidity in animals but also the scrutiny of tissue healing following the administration of antibiofilm drugs (Fitzgerald et al., 2017; Choi et al., 2020; Silveira et al., 2021; Farshadzadeh et al., 2022). Notably, investigations employing catheters as subcutaneous implants and inducing *S. aureus* infections have revealed elevated CFU counts in subjects with catheters, concomitant with a concurrent diminishment in tissue pathology. This observed phenomenon is ascribed to the catheter’s role in constraining the dissemination of bacteria through the adjacent tissues (Jordan et al., 2022).

*In vivo* models for staphylococcal infections originating from the deployment of medical devices facilitate the investigation of biofilm development across varied materials and in unique infection scenarios, encompassing conditions such as prosthetic joint infections or catheter-related infections (Carli et al., 2017).

We employed a straightforward experimental model wherein an early biofilm formation inhibitor was introduced into subcutaneous skin pouches already harboring infections initiated by either *S. aureus* or *P. aeruginosa*, known for their heightened biofilm production. This model offers a streamlined interpretation as it involves only two variables: bacterial multiplication coupled with biofilm formation and the efficacy of the biofilm inhibitor. Burkatovskaya et al. (2008) undertook analogous experiments using an *in vivo* mouse wound infection model, infiltrating chitosan acetate into a wound dressing (Burkatovskaya et al., 2008). Despite the bandage application leading to enhanced wound healing, challenges arose, such as mice dislodging the bandages or encountering difficulties during the experiment’s initial stages of bandage removal.

Our *in vivo* model exhibited no discernible morbidity in mice, suggesting a localized infection. It is crucial to emphasize that PDIA does not impact bacterial multiplication but selectively hinders biofilm production. The comparison of staphylococcal multiplication in the wound with clinical symptoms and histology may indicate that the bacteria, although multiply in the wound, are unable to build biofilm and are more readily ingested by the accumulated phagocytic cells. Introducing a foreign body into a wound inevitably complicates the experimental course of infection (particularly with *S. aureus*), elevating bacterial virulence and multiplication rates (Carli et al., 2017; Jordan et al., 2022). Despite the absence of significant differences in clinical observations, the relatively short experimental duration (72 h) might have influenced the outcomes. Notably, even under heightened bacterial doses causing substantial stress to the mice, no fatalities occurred in any of the study groups. In a related study, Lu et al. investigated a mouse wound infection model, exploring the impact of varying inoculum concentrations of infectious *S. aureus* over a two-day infection period, encompassing bacterial growth and subclinical effects. Results revealed diminished bacterial numbers at the minimum concentration of 10^6^ CFU/mL, accompanied by a broader spectrum of infection signs. Conversely, administering a higher quantity of bacteria (specifically 10^7^ CFU/mL) led to a noteworthy enhancement in the consistency of subclinical effects (Lu et al., 2014). The primary focus, therefore, centered on microbiological and histopathological parameters, enabling a comprehensive analysis of tissue dynamics (Versey et al., 2021).

Thus, our findings on *S. aureus* 48 suggest a tangible impact of PDIA on the organization and structure of *S. aureus* 48 populations within the wound. This effect may exhibit synergy with the actions of certain antibiotics, particularly those impeding cell wall synthesis. Instances of favorable interactions between biofilm inhibitors and antibiotics are documented in the literature (Pletzer et al., 2016; Roy et al., 2018; Silveira et al., 2021), and are supported by *in vitro* studies (Cirioni et al., 2006; Donelli et al., 2007; Kaplan et al., 2012; Ma et al., 2017; Pletzer et al., 2018). These interactions are worthy of being investigated in future studies on PDIA.

Tissue granulation found in biofilm infections can indicate ongoing acute inflammation, often accompanied by infiltrating inflammatory cells (neutrophils). Pro-inflammatory molecules secreted by neutrophils can lead to the destruction of host cells (Gajula et al., 2020; Versey et al., 2021; Murphy et al., 2023).

Furthermore, biofilm-associated wound infections involving *S. aureus* induce abnormalities in the collagen composition of granulation tissue, thereby resulting in compromised biomechanical properties of the wound tissue (Gurjala et al., 2011; Roy et al., 2020). The outcomes of our investigation substantiate this postulate; the extent of tissue granulation within the *Staphylococcus*-infected group (*S. aureus* 48 group) exhibited the highest magnitude, whereas the administration of PDIA iminosugar significantly attenuated this histopathological parameter (*S. aureus* 48 + I group). Analogously, a parallel deduction can be drawn for neutrophil infiltration, with PDIA iminosugar prominently diminishing the levels thereof in the context of staphylococcal infection. Comparable findings were elucidated by Zaleski et al. (2006) in examining the impact of hyaluronic acid-binding peptides on staphylococcal infection in mouse wounds. Despite manifesting no antimicrobial efficacy *in vitro*, specific peptides demonstrated noteworthy *in vivo* effectiveness by diminishing bacterial burden and ameliorating inflammation in mouse wounds (Zaleski et al., 2006).

Zaleski et al. investigated whether the hyaluronic acid-binding peptide PEP35, which exhibited the most efficacious *in vivo* response against staphylococcal infection in mouse wounds, could modulate the host inflammatory response *in vivo.* The investigation showcased the immunomodulatory prowess of PEP35, evident in the augmentation of CXC cytokine production, attracting neutrophils to the infection site (Zaleski et al., 2006). Furthermore, the brief half-life of PEP35 precluded a protracted inflammatory response that might result in tissue damage (Lee et al., 2010).

In addition to mitigating bacterial numbers and ameliorating histopathological parameters during staphylococcal infection in murine wounds, as observed with the tested PDIA iminosugar, it also demonstrated a reduction in inflammation in the non-infected wound itself, as evidenced by the tissue granulation results in the control groups (Control vs. Control + I). Thus, PDIA iminosugar exerts an antibiofilm effect and potentially possesses immunomodulatory properties. Further exploration of this aspect would be advantageous in subsequent research endeavors.

In the context of wounds afflicted with *Pseudomonas* infection, a discernible impediment to the healing process is evident, as documented by other researchers (Zhao et al., 2010; Seth et al., 2012; Watters et al., 2013). Our investigation, however, did not reveal indications of an augmented inflammatory response or a significant microbial burden in the groups infected with *P. aeruginosa* 5. The inefficacy of the inhibitor in *P. aeruginosa* mice infection remains a perplexing observation, especially considering the pronounced *in vitro* efficacy of the PDIA iminosugar (Kozień et al., 2022). In contrast to *S. aureus*, the employed *P. aeruginosa* strain might have been ineffectual in establishing and perpetuating the infection within the wound, or perhaps the infectious dose employed was insufficient.

To offer a plausible elucidation, experiments were conducted to juxtapose the *in vitro* effects of H_2_O_2_, serving as an exemplar of an inflammatory oxygen burst, on the bacteria utilized in the *in vivo* studies. The findings indicated that *P. aeruginosa* 5 exhibited greater susceptibility to the adverse effects of H_2_O_2_ than *S. aureus* 48. This susceptibility may be contingent upon the specific strain. The amelioration of oxidative stress in *P. aeruginosa* is contingent upon the expression of genes such as superoxide dismutase (SOD), catalase (KatA, and KatB) (Hassett et al., 1999). Conversely, in the case of *S. aureus*, the PerR regulator members, alkyl hydroperoxide reductase (AhpC), and KatA are the pertinent factors (Cosgrove et al., 2007). These observations explicate the histopathological disparities observed between staphylococcal and *Pseudomonas* infections in the mice under examination. The *Pseudomonas* strain was probably effectively eliminated from the inflamed tissue through reactive oxygen species formed during tissue inflammation (Winterbourn et al., 2016).

As evinced above, the PDIA biofilm inhibitor manifests *in vivo* efficacy in a wound infection model in mice with *S. aureus*, thereby prompting the initiation of more sophisticated experiments, including those involving foreign bodies in conjunction with selectively chosen antibiotics.

## Data availability statement

The original contributions presented in the study are included in the article/**Supplementary Material**. Further inquiries can be directed to the corresponding author.

## Ethics statement

The animal study was approved by Second Local Institutional Animal Care and Use Committee (IACUC) in Kraków. The study was conducted in accordance with the local legislation and institutional requirements.

## Author contributions

ŁK: Data curation, Investigation, Methodology, Project administration, Validation, Writing – original draft, Writing – review & editing. AP: Data curation, Investigation, Writing – review & editing. PH: Conceptualization, Funding acquisition, Supervision, Writing – original draft. ZA: Investigation, Methodology, Resources, Writing – review & editing. UB: Investigation, Writing – review & editing. LP: Investigation, Writing – review & editing. AP-F: Investigation, Writing – review & editing. EG: Resources, Writing – review & editing. PP: Formal analysis, Writing – review & editing. KO: Investigation, Writing – review & editing. AT-P: Methodology, Writing – review & editing, Data curation, Formal analysis. MS: Conceptualization, Methodology, Supervision, Writing – review & editing.

## Supplementary material

Supplementary Figure 1 Box plot showing the distribution of clinical observations (wound healing, edema, redness, exudate) conducted on infected, treated, and control animals. The graph indicates statistical significance obtained after comparing all results.
