# Supplementary figures and images for: PDIA iminosugar influence on subcutaneous Staphylococcus aureus and Pseudomonas aeruginosa infections in mice
Source: Front Cell Infect Microbiol. 2024 Jul 31;14:1395577. doi: 10.3389/fcimb.2024.1395577 (PMC11322076; doi:10.3389/fcimb.2024.1395577)

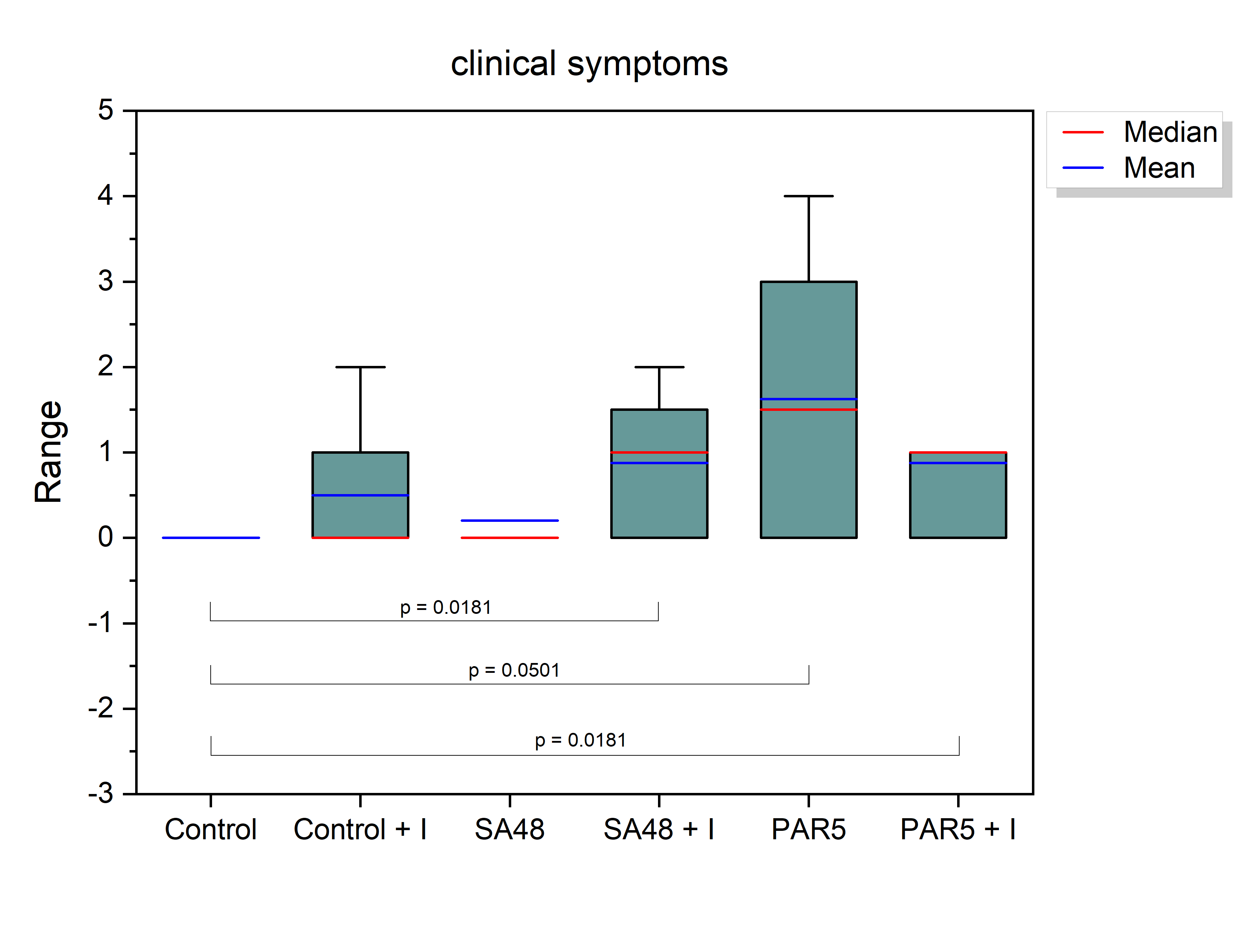

Supplement: Supplementary Figure 1 — Box plot showing the distribution of clinical observations (wound healing, edema, redness, exudate) conducted on infected, treated, and control animals. The graph indicates statistical significance obtained after comparing all results. [file Image_1.tif]
